# Comparative Sensitivity and Specificity of the 7SL sRNA Diagnostic Test for Animal Trypanosomiasis

**DOI:** 10.3389/fvets.2022.868912

**Published:** 2022-04-05

**Authors:** Maria Contreras Garcia, Emily Walshe, Pieter C. Steketee, Edith Paxton, Javier Lopez-Vidal, Michael C. Pearce, Keith R. Matthews, Fatima Ezzahra-Akki, Alec Evans, Karen Fairlie-Clark, Jacqueline B. Matthews, Finn Grey, Liam J. Morrison

**Affiliations:** ^1^Roslin Institute, Royal (Dick) School of Veterinary Studies, University of Edinburgh, Edinburgh, United Kingdom; ^2^Roslin Technologies Limited, Roslin Innovation Centre, University of Edinburgh, Edinburgh, United Kingdom; ^3^Ashworth Laboratories, Institute of Immunology and Infection Research, School of Biological Sciences, University of Edinburgh, Edinburgh, United Kingdom; ^4^Global Alliance for Livestock Veterinary Medicines, Edinburgh, United Kingdom; ^5^Clinvet Morocco, Mohammedia, Morocco

**Keywords:** animal trypanosomiasis, diagnostic, small RNA, sensitivity, specificity

## Abstract

Animal trypanosomiasis (AT) is a significant livestock disease, affecting millions of animals across Sub-Saharan Africa, Central and South America, and Asia, and is caused by the protozoan parasites *Trypanosoma brucei, Trypanosoma vivax, and Trypanosoma congolense*, with the largest economic impact in cattle. There is over-reliance on presumptive chemotherapy due to inadequate existing diagnostic tests, highlighting the need for improved AT diagnostics. A small RNA species, the 7SL sRNA, is excreted/secreted by trypanosomes in infected animals, and has been previously shown to reliably diagnose active infection. We sought to explore key properties of 7SL sRNA RT-qPCR assays; namely, assessing the potential for cross-reaction with the widespread and benign *Trypanosoma theileri*, directly comparing assay performance against currently available diagnostic methods, quantitatively assessing specificity and sensitivity, and assessing the rate of decay of 7SL sRNA post-treatment. Results showed that the 7SL sRNA RT-qPCR assays specific for *T. brucei, T. vivax, and T. congolense* performed better than microscopy and DNA PCR in detecting infection. The 7SL sRNA signal was undetectable or significantly reduced by 96-h post treatment; at 1 × curative dose there was no detectable signal in 5/5 cattle infected with *T. congolense*, and in 3/5 cattle infected with *T. vivax*, with the signal being reduced 14,630-fold in the remaining two *T. vivax* cattle. Additionally, the assays did not cross-react with *T. theileri*. Finally, by using a large panel of validated infected and uninfected samples, the species-specific assays are shown to be highly sensitive and specific by receiver operating characteristic (ROC) analysis, with 100% sensitivity (95% CI, 96.44–100%) and 100% specificity (95% CI, 96.53–100%), 96.73% (95% CI, 95.54–99.96%) and 99.19% specificity (95% CI, 92.58–99.60%), and 93.42% (95% CI, 85.51–97.16% %) and 82.43% specificity (95% CI, 72.23–89.44% %) for the *T brucei, T. congolense* and *T. vivax* assays, respectively, under the conditions used. These findings indicate that the 7SL sRNA has many attributes that would be required for a potential diagnostic marker of AT: no cross-reaction with *T. theileri*, high specificity and sensitivity, early infection detection, continued signal even in the absence of detectable parasitaemia in blood, and clear discrimination between infected and treated animals.

## Introduction

Animal trypanosomiasis (AT) is an infectious disease caused by single cell protozoan parasites of the *Trypanosoma* genus (*Trypanosoma brucei, Trypanosoma congolense* and *Trypanosoma vivax*), which are cyclically transmitted by tsetse flies (*Glossina sp*.), or, in the case of *T. vivax and Trypanosoma brucei evansi*, mechanically transmitted by biting flies. The disease is found across Sub-Saharan Africa (*T. brucei, T. congolense* and *T. vivax*), South America (*T. vivax and T. b. evansi*) and North Africa and Asia (*T. b. evansi*), where many domestic livestock types (cattle, sheep, goats, equids, camels and pigs) are susceptible to infection, although the heaviest economic and welfare burden of the disease is considered to fall on cattle (1).

AT is considered a major constraint on agricultural production in Sub-Saharan Africa; it is estimated to infect over 70 million cattle annually and is responsible for production losses that total billions of dollars per year (2, 3). *T. vivax* is a cause of concern in Central and South America where its geographical distribution has grown in recent years (4–6). Current estimates indicate that over 11 million cattle are at risk of infection in the extensive cattle ranching areas of the Brazilian Pantanal and Bolivian lowlands alone (5). Similarly, *T. b. evansi*, a mechanically transmitted variant of *T. brucei*, causes disease across Asia in equids, camels, cattle and water buffalo, and is also present in South America as a pathogen of increasing concern to the cattle industry (7, 8).

Cattle infected by trypanosomes show non-specific clinical signs such as fever, anemia and weight loss, and usually develop a chronic infection with periods of high parasite blood load followed by periods of sub-patent parasitaemia. Symptoms are usually aggravated if animals are overworked and malnourished, which can contribute to high levels of mortality (9). The disease is also linked to lowered milk production, reduced working power and reproductive failure (3, 10). Moreover, the immunomodulatory effects of AT may adversely affect the efficacy of vaccines (11), treatments (12), and the immune response and outcome to co-infecting pathogens (13).

Diagnosis of AT can be performed by detection of the parasite itself, the parasite's DNA or antibodies against specific parasite antigens. In the field currently, diagnosis is mainly undertaken by visualization of trypanosomes in blood. The simplest forms of visual detection are by observation of wet or Giemsa-stained blood films, but these have low sensitivity. Improvements on these methods are the microhaematocrit centrifugation technique (MHCT) and buffy coat technique (BCT), which concentrate the blood fraction where trypanosomes are found, thus increasing sensitivity. These microscopy techniques have the advantage of being relatively simple to perform and are low cost, but have relatively poor sensitivity, especially when parasite blood load is low (a minimum of 2.5 × 10^2^ parasites/mL of blood is needed for detection by BCT and MHCT) (14, 15). Molecular techniques such as PCR are highly sensitive and are reported to detect parasites loads as low as 1 parasite/mL of blood (16). However, the application of PCR diagnostics requires experienced personnel and equipment. Either PCR or indirect ELISA are the diagnostic methods of choice for epidemiological studies on *Trypanosoma sp*. due to their high sensitivity and potential for high throughput. While there is one commercial ELISA available (17), due to the presence of long-lasting antibodies ELISA can be poor at distinguishing between exposure and ongoing infection, making PCR the preferred method to avoid overestimating the prevalence of the disease.

Since field-applicable diagnostic methods have relatively poor accuracy and veterinary support is not always available, disease control largely focuses on chemotherapy or chemoprophylaxis following presumptive diagnosis (18, 19). However, there are only four available trypanocidal drugs [diminazene aceturate, homidium salts and isometamidium chloride are the most widely used for cattle, sheep and goat infections, and quinapyramine salts for *T. b. evansi* infections, particularly in equids (3)], to which resistance is becoming increasingly common due to inappropriate use; this is compounded by the fact that no new trypanocidal actives have been approved in the last 60 years (3, 19). Vector control is also deployed as a control measure, but this is difficult to achieve successfully due to the range and mobility of the tsetse fly; indeed, sustainable removal has been achieved in less than 2% of the infested territory (20). Better diagnostics are essential to ensure the efficacy of the few available trypanocides is preserved through effective and sustainable use (19). In addition, better estimates of disease prevalence through more accurate diagnosis would represent a first step toward establishing appropriate and sustainable control activities (18).

Besides the AT-causing trypanosomes, other trypanosome species can infect livestock. The parasite *Trypanosoma* (megatrypanum) *theileri* is prevalent in cattle throughout the world with surveys indicating a presence in >80% of cattle in the US, Europe and the United Kingdom (21–25). In Sub-Saharan Africa, *T. theileri* is highly prevalent in the cattle population and coexists with AT-causing trypanosomes (14, 26, 27). *T. theileri* is transmitted by tabanid flies and is considered a commensal pathogen. Clinical disease due to this parasite is infrequent in healthy cattle and parasite levels are usually low in blood and tissues, being limited by the hosts' immune system. Infection with *T. theileri* is believed to be lifelong (21, 28). The coexistence of African trypanosomes with the benign *T. theileri* makes distinction between the trypanosome species desirable to avoid unnecessary treatment of cattle.

In a recent study (29) we described the presence of a parasite-specific small RNA derived from the 7SL RNA. The small RNA is secreted/excreted by trypanosomes and is present at high concentrations in the blood of infected cattle, making it a potential marker of active infection, even in periods of sub-patent parasitaemia. In addition, 7SL sRNA presents sequence differences between the three main trypanosome species (*T. brucei, T. congolense and T. vivax*) supporting the development of a species-specific RT-qPCR based test.

The 7SL sRNA has also been found to be a sensitive diagnostic marker for equine infections with *Trypanosoma brucei equiperdum*, the causative agent of dourine; 7SL sRNA signal was detected in horses experimentally infected with *T. b. equiperdum* before parasite detection by microscopy, and earlier than seroconversion detection using a complement fixation test (CFT), which is the officially-recommended dourine test by OIE (30). The 7SL sRNA signal remained present in periods of sub-patent parasitaemia but decayed rapidly after trypanocidal treatment, indicating that presence of the marker correlated with active infection and that the test is specific (i.e., correctly identifies absence of infection as negative) (31). Verney et al. also showed that 7SL sRNA is stable at 30°C for 7 days; a highly desirable characteristic for the application of the test in areas with limited or no cold chain capabilities.

Here, we explore further the detection of 7SL sRNA using RT-qPCR as a diagnostic method for AT. We compare the performance of the test with parasite detection in blood and DNA PCR from the same cattle, we describe the dynamics of 7SL sRNA decay after treatment with trypanocides, assess the potential for cross-reaction with *T. theileri*, and analyse the sensitivity and specificity of this diagnostic method.

## Materials and Methods

### Ethics Statement

Animal experiments were carried out at the Roslin Institute, University of Edinburgh under the auspices of Home Office Project License number 60/4394. Studies were approved by the Roslin Institute Animal Welfare and Ethical Review Board (study numbers L412 and L424). Care and maintenance of animals complied with University regulations and the Animals (Scientific Procedures) Act (1986; revised 2013). Protocol plans for studies carried out at Clinvet were submitted to the Institutional Animal Care and Use Committee (IACUC), which issued certificates of approval. The protocol was designed to allow the use of the study animals in compliance with the Clinvet policy on the ethical use of animals, according to the specifications in Directive 2010/63/EU of the European Parliament and of the Council on the protection of animals used for scientific purposes. Approved study number: CG626-CVM19/345.

### *In vivo* Infections

Samples from experimentally infected cattle were obtained from two sites.

Site 1: Experimental infections of cattle with *T. congolense* IL3000 and *T. brucei* Antat 1.1 were conducted in the vector-proof animal facilities of the Roslin Institute. Post-weaning male Holstein-Friesian cattle (*n* = 4 per each trypanosome species) were inoculated intravenously via the jugular vein with 1 × 10^6^ trypanosomes, and infections followed for 28 days. Blood sampling typically occurred every 2 days. At day 28, all calves were treated with an intramuscular dose of diminazene aceturate (CEVA, France; 7 mg/kg body weight) and monitored for a further 96 h, sampling every 24 h, to assess infection clearance and signal decay of the 7SL sRNA. Parasitaemia was typically measured every 2 days in jugular blood samples by using the buffy coat technique (15, 32). Whole blood samples were also snap-frozen in liquid nitrogen to facilitate DNA extractions at later points, although for the *T. brucei* infections this was in error only initiated at day 15 post-infection. Serum was collected at each sampling time point to measure the 7SL sRNA signal; serum was extracted from 10 mL blood collected in red top (clot activator) vacutainer tubes. Briefly, blood tubes were incubated at 37°C for 1 h, then allowed to clot at room temperature overnight. The following day, red blood cells were removed by spinning the tubes at 1500 × *g* for 15 min, serum was aliquoted and stored at −80°C until use. For *T. theileri*, eight Holstein-Friesian calves (6-weeks old) were injected intravenously with 1 × 10^7^ *T. theileri* parasites [Edinburgh strain (21, 28)] and infections were followed for 8 weeks. 10 mL blood samples were taken at weekly intervals into EDTA-containing tubes (Becton-Dickinson, US) and DNA extracted. Ten mL blood was also collected in red top (clot activator) collection tubes; after collection the sample was left undisturbed at room temperature for 30 min, and was then centrifuged at 1400 × *g* for 10 min at 4°C. After centrifugation, aliquots were prepared and stored at −20°C until use.

Site 2: Cattle housed in vector-proof facilities at Clinvet Morocco were experimentally infected with *T. congolense* Kont2/133 (*n* = 15) or *T. vivax* STIB 719 (*n* = 15). Post-weaning male Holstein-Friesian were inoculated intravenously via the jugular vein with 1 × 10^6^ trypanosomes. For each trypanosome species cattle were separated into three treatment groups of five and treated with an experimental drug when their parasitaemia reached the peak (score ≥4, equivalent to approximately 10^4^ – 5 × 10^5^ parasites/ mL), or if there was a >20% reduction from the previous day's packed cell volume (PCV). Samples were collected 8 days before infection, at day 0 (prior to parasite infection), and typically every 2 days after infection; 10 mL blood was collected by jugular venepuncture into EDTA tubes, and plasma prepared by centrifuging at 1500 × *g* for 10 min at 4°C and removing the supernatant (= plasma). After treatment, parasitaemia was monitored for a further 96 h, and blood samples collected for plasma preparation every 24 h, to assess infection clearance.

### Parasitaemia Measurements

Blood samples were analyzed for presence of parasites using the buffy coat technique, and parasitaemia scored based on the number of trypanosomes observed in each preparation (32).

### RNA Extraction

RNA extractions were performed using Trizol LS (Invitrogen, US), following the manufacturer's instructions, with an additional 75% ethanol wash step. In total 250 μL of serum or plasma from experimentally infected cattle from sites 1 and 2 were used as the starting material for each RNA extraction.

### Reverse Transcription Quantitative PCR (RT-qPCR)

RT-qPCR was performed with the previously described species-specific 7SL-derived small RNA stem loop primer-probe detection assays (Thermo Fisher, UK, Custom TaqMan Small RNA assay catalog number 4398989 [assay IDs *T. brucei*: CTFVKNM; *T. congolense*: CTRWEM9; *T. vivax*: CTDJXGZ]) (29). Reverse transcription was undertaken using a cDNA Reverse Transcription Kit (Applied Biosciences, US) following the manufacturer's instructions but replacing the random primers with the above-mentioned TaqMan assay RT primer. Three μL of RNA, extracted from serum or plasma, were used per each 15 μL reaction. The following thermocycling conditions were applied for the RT reaction: 16°C for 30 min, 42°C for 30 min, and a final step at 85°C for 5 min to inactivate the reverse transcriptase. After the RT reaction, qPCR was performed using a commercial kit (TaqMan universal PCR master mix, Thermo, UK), according to the manufacturer's instructions. One μL custom primer-probe, along with 1.5 μL RT reaction product, was also added per 20 μL reaction. The qPCR cycling profile was as follows: 50°C for 2 min, 95°C for 10 min and 40 cycles at 95°C for 15 seconds and 60°C for 1 min (probe-detection step). Relative 7SL sRNA expression (dCt) was calculated by normalizing to an uninfected serum control. For each qPCR reaction, negative controls included RT negative samples and no template controls, and positive controls were samples extracted from pure cultures of *T. brucei* or *T. congolense*, or infected blood in the case of *T. vivax*.

### DNA Extraction

For whole blood samples from cattle infected with *T. brucei* and *T. congolense* at Roslin, DNA extraction was performed using a commercial kit (DNeasy blood and tissue kit, Qiagen, UK) following the manufacturer's instructions for non-nucleated blood samples. Whole blood (100 μL) was used as the starting material for the extractions and DNA was eluted in 50 μL nuclease-free water. Note that for the *T. brucei* infections, due to error whole blood for DNA was only collected from day 15 post-infection onwards. For whole blood samples taken from cattle infected with *T. theileri*, DNA was extracted as follows. One mL blood was mixed thoroughly with 1 mL RBC lysis buffer (0.32 M sucrose, 10 mM Tris-HCl pH 7.5, 5 mM MgCl2, 0.75% Triton X-100) in a microfuge tube. The samples were then centrifuged at 18,000 × *g* for 1 min to pellet all cells and the supernatant was removed. The pellets were repeatedly resuspended and recovered from 1 mL aliquots of RBC lysis buffer until no red blood cells were present. The resulting pellets were resuspended in 100 μL lysis buffer (50 mM KCl, 10 mM Tris-HCl pH 8.3, 2.5 mM MgCl2, 0.1 mg/mL gelatin, 0.45% NP40, 0.45% Tween-20, 60 μg/mL proteinase K) and kept at 55°C overnight. The samples were then extracted with phenol:chloroform:isoamyl alcohol (Sigma, UK) prior to storage at −20°C until use.

### PCR

DNA PCR was performed using the previously described primers TBR (for *T. brucei*) (33), and TCS (for *T. congolense* Savannah) (33). The PCR reaction was performed using the Quick-load Taq 2x master mix (NEB, UK) following the manufacturer's instructions. One μL DNA, 1 μL 10 μM (forward and reverse) primer, and 12.5 μL “quick load” mastermix were added per 20 μL reaction. PCR cycling conditions were 30 cycles at 95°C for 50 seconds, 60°C for 50 seconds and 65°C for 60 seconds. PCR products were visualized in a 1% agarose gel containing 1:10000 gelRed dye.

Nested PCR reactions were designed to amplify the *T. theileri* β-α tubulin intergenic sequence or the SSU rRNA gene to identify any *T. theileri* population. The primers used were as follows: TUB F1 (5′-AGTAGCAACGACAGCAGCAGT-3′), TUB R1 (5′-GTAAAGTGTTTGAAGAAGAGCTCG-3′), TUB F2 (5′-CGATTCTCTTCGCCTGTTTGT-3′), TUB R2 (5′-ACTAACCGCGACCAAAGAAGT-3′), SSU rRNA F1 (5′- AGTGATGGCCGTGTAGTGGAC-3′), SSU rRNA R1 (5'- GAGGCGAGGAGCGAGATGAA-3′), SSU rRNA F2 (5′- GGAGAGGGAGCCTGAGAAATAGC-3′) and SSU rRNA R2 (5'- GCACGACGCCATAATCTCCAATG-3′). Each 25 μL reaction contained the following components: 5 μL template, 1x PCR buffer, 0.2 mM of each dNTP, 1.25 mM MgCl2, 0.4 μM of each primer and 0.25 U Phire Green Hot Start II (Thermo Scientific, US). The first round PCR reactions were heated to 98°C for 30 seconds, followed by 35 cycles of denaturation at 98°C for 5 seconds, annealing at 60°C for 45 seconds and elongation at 72°C for 45 seconds. Following the final cycle, the reactions were extended for a further 1 min. The second round nested PCR reaction was conducted using the same conditions with 5 μL of the first reaction diluted 1:50 as template.

### Data Analysis

Population genomics analysis of the 7SL-sRNA: to analyse conservation of the 7SL-sRNA within trypanosome species, genome sequencing data was retrieved from previous population genomic studies of *T. brucei* [(34); 85 samples], *T. congolense* [(35); 51 samples] and *T. vivax* [(36); 27 samples]. For *T. congolense* and *T. vivax*, raw data was aligned to the respective genomes [TriTrypDB v56.0; Tcongolense_2019 and Tvivax_Y486 genomes for *T. congolense* and *T. vivax*, respectively (37)] using HiSat2 (–no-spliced-alignment –k), and alignments were filtered using samtools (-q 1 –F 0 × 100) to generate aligned bam files. Summaries of mapped reads were generated using the samtools mpileup utility and data were analyzed for variants using the bcftools package. A consensus sequence was generated in fasta format (bcftools, “consensus” function) for the genomic region encompassing the 7SL RNA (*T. congolense*: pschr_08:759706-759800; *T. vivax*: TvY486_08:701470-701550) and sequences were aligned using CLC Genomics Workbench. For analysis of *T. brucei*, the “Search SNPs by Gene IDs” tool was employed in TriTrypDB (v56.0) (37), using the full length 7SL RNA gene (Tb927.8.2861), and sequences for 85 isolates from Weir et al. (34) were imported into CLC Genomics Workbench for alignment. Alignments were generated using the 7SL sRNA sequence only. The *T. theileri* 7SL sRNA was identified by blasting the *T. brucei* TREU 927 7SL RNA sequence (Tb927.8.2861; TriTrypDB v56.0) against the *T. theileri* genome [Edinburgh isolate (21)] followed by visual identification of the 26-bp 7SL sRNA based on the resulting sequence alignment created in CLC Genomics Workbench.

Receiver operating characteristic (ROC) curves were calculated using GraphPad Prism software v.8.4. Ct values of the RT-qPCRs, normalized to a serum control from an uninfected cow, were used as input for the ROC analysis. Samples were classified as uninfected if they were taken before parasite inoculation. Samples were considered infected from the time of inoculation up until treatment. ROC curves were calculated using the Wilson/Brown method (38) with a 95% confidence interval. The cut-off point was evaluated using two commonly used methods. Firstly, the decision for optimal sensitivity and specificity was based on finding the cut-off point yielding the minimal value for (1 – sensitivity)^2^ + (1 – specificity)^2^, which is the point of the curve closest to the (0,1) coordinate (defined as (1 – specificity=0) and sensitivity =1), a point that would represent the perfect test (39). The second method used was Youden's index (J), which defines the optimal cut-off as the one that maximizes (sensitivity + specificity – 1). In the graph, J is calculated as the point of the curve with the greatest vertical distance to the diagonal line (40).

## Results

### Comparison of Diagnostic Methods

An important step in the evaluation and characterization of new diagnostic tests is to investigate whether the test presents an improvement over the accuracy of existing diagnostic methods. However, there is a lack of comparative studies evaluating diagnostics accuracy using samples that derive from well-defined trypanosome infections of cattle. The availability and use of such samples would allow for a more reliable evaluation and comparison of the tests' accuracy.

To compare the diagnostic accuracy and sensitivity of the 7SL sRNA RT-qPCR to currently available AT diagnostic methods, samples from the same experimental infections were tested by BCT, PCR on DNA extracted from whole blood, or 7SL sRNA RT-qPCR on RNA extracted from serum/plasma. Holstein-Friesian calves were experimentally infected with *T. brucei* Antat 1.1 or *T. congolense* IL3000 (*n* = 4 per species) and infections followed for 28 days. Successful establishment of the infection in all calves was confirmed by parasite detection in blood by microscopy 3–5 days after the initial inoculation. After 28 days, all calves were treated with the trypanocide diminazene aceturate, and sampled 24-, 48-, 72- and 96-h post-treatment.

Blood samples were tested for trypanosome infection with the BCT, PCR (TBR or TCS primers) or 7SL sRNA RT-qPCR. When using the BCT for diagnosis, samples that showed presence of trypanosomes were given a score from 1 to 6 (positive), whereas absence of visible trypanosomes was scored as 0 (negative) (15). Approximate parasitaemia equivalences (trypanosomes/mL) of the score used are: 1 = 1 × 10^2^; 2 = 1 × 10^3^; 3 = 5 × 10^3^ – 5 × 10^4^; 4 = 1 × 10^4^-5 × 10^5^; 5 = 5 × 10^5^ −5 × 10^6^; 6 = >5 × 10^6^. In PCR, the presence of a band of the expected size (TBR= 177 bp, TCS= 316 bp) was considered a positive result for trypanosome infection. The 7SL sRNA signal for any post-infection time point was compared to the baseline established with the sample pre-infection, an increase in 7SL sRNA signal was considered positive.

### *Trypanosoma brucei* Infections

**Figure 1:**
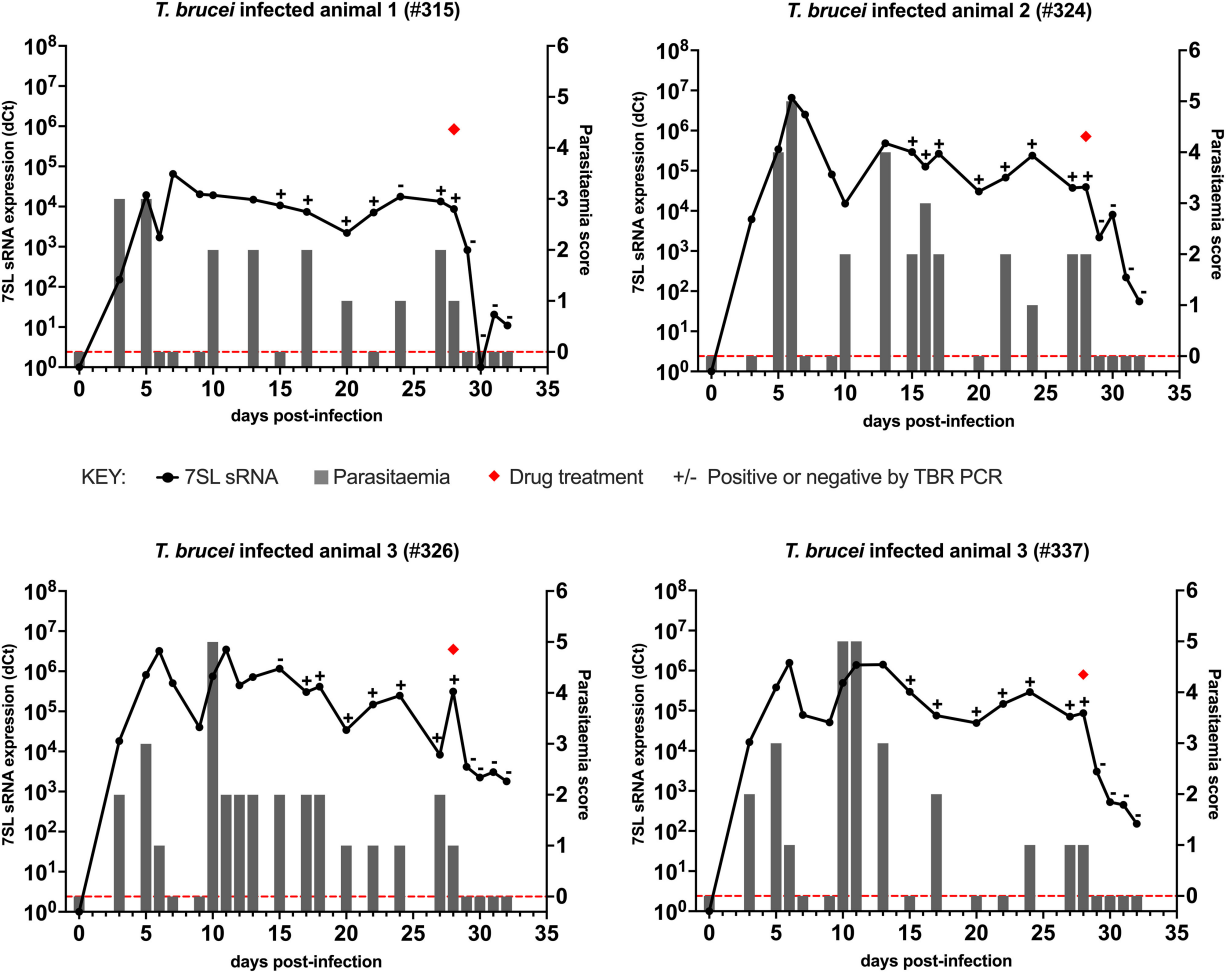
Comparison of detection by buffy coat microscopy, TBR PCR and 7SL sRNA RT-qPCR in *T. brucei* infected calves. Four calves (Holstein-Friesian, approximately 4–6 months old) were infected with *T. brucei* Antat 1.1 (day 0) and monitored for 32 days. On day 28, calves were treated with diminazene aceturate, indicated by a red diamond. Parasitaemia (right y axis) was determined on average every 2 days by the BCT; days on which this was measured are indicated by gray bars. Approximate parasitaemia equivalences (trypanosomes/mL) of the score used: 1 = 1 × 10^2^; 2 = 1 × 10^3^; 3 = 5 × 10^3^-5 × 10^4^; 4 = 1 × 10^4^-5 × 10^5^; 5 = 5 × 10^5^-5 × 10^6^; 6 = >5 × 10^6^. Gray bars measuring 0 indicate time points in which parasitaemia was measured but no parasites were detected (0 on y axis is shown by red dotted line). Relative expression (dCt) of 7SL sRNA by RT-qPCR (left y axis) is shown with a black line and was calculated by normalizing to an uninfected control animal. TBR PCR results are shown by plus (+) or minus (–) signs indicating the presence or absence of the expected PCR band on an agarose gel, respectively.

In cattle infected with *T. brucei* Antat 1.1 (Figure 1), using BCT, parasites were detected 3 days after infection in three out of the four cattle, whereas using the 7SL sRNA RT-qPCR parasite infection was detected in all four cattle. After initial detection, the 7SL sRNA signal remained detectable for the duration of the infection, whereas the ability of the BCT to detect parasite presence fluctuated. BCT parasite detection after infection ranged from 9/14 to 15/17 of the sampled time points per animal. From day 15 post-infection, samples were also tested with the TBR-PCR (unfortunately appropriate samples were not collected before this timepoint), this method identified the presence of parasites in all but two samples (315 day 24 and 326 day 15). After treatment with diminazene aceturate (day 29 to 32), BCT and TBR-PCR were not able to detect parasite presence, suggesting a rapid signal decay. However, the 7SL sRNA signal decreased on average by 563-fold over 96 h but remained detectable, suggesting the small RNA was still present in circulation.

Overall, the use of the three diagnostic methods to follow the course of infection revealed that the molecular methods (TBR PCR and 7SL sRNA RT-qPCR) were able to detect infection in periods of sub-patent infection, indicating their increased sensitivity compared to currently available methods. Signal decay after treatment was faster when monitored with the BCT and PCR compared to the 7SL sRNA RT-qPCR.

### *Trypanosoma congolense* Infections

**Figure 2:**
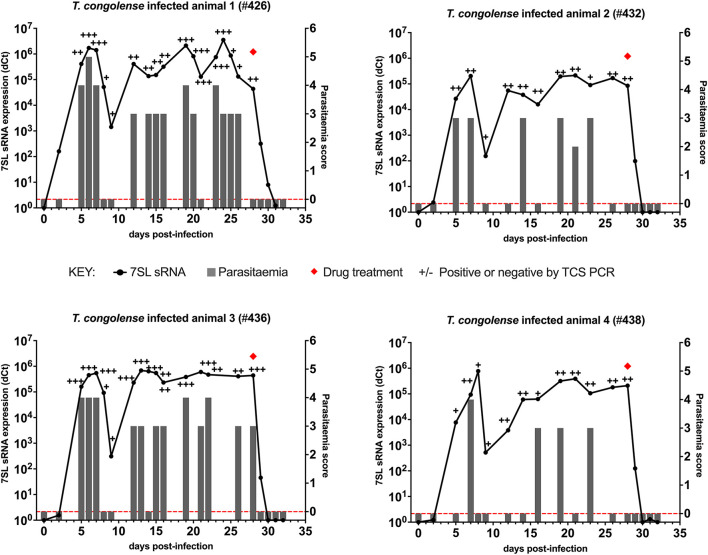
Comparison of detection by buffy coat microscopy, TCS PCR and 7SL sRNA RT-qPCR in *T. congolense* infected calves. Four calves (Holstein-Friesian, approximately 4–6 months old) were infected with *T. congolense* IL3000 (day 0) and monitored for 32 days. On day 28, calves were treated with diminazene aceturate, indicated by a red diamond. Parasitaemia (right y axis) was determined on average every 2 days by the BCT and is indicated by gray bars. Approximate parasitaemia equivalences (trypanosomes/mL) of the score used: 1 = 1 × 10^2^; 2 = 1 × 10^3^; 3 = 5 × 10^3^-5 × 10^4^; 4 = 1 × 10^4^-5 × 10^5^; 5 = 5 × 10^5^-5 × 10^6^; 6 = >5 × 10^6^. Gray bars measuring 0 indicate time points in which parasitaemia was measured but no parasites were detected (0 on y axis is shown by red dotted line). Relative expression (dCt) of 7SL sRNA by RT-qPCR (left y axis) is shown with a black line and was calculated by normalizing to an uninfected control animal. TCS PCR results are shown by plus (+) or minus (–) signs indicating the presence or absence of the expected band on an agarose gel, respectively.

In cattle infected with *T. congolense* IL3000 (Figure 2), 7SL sRNA signal was first detected 2 days after inoculation in all four cattle using 7SL sRNA RT-qPCR, whereas parasites were first detected 5 days after infection using TCS PCR and BCT. After initial detection, 7SL sRNA remained detectable throughout the duration of the infection period (28 days) in all four cattle. The TCS PCR remained positive after the initial detection in all four cattle. The BCT, however, was not able to consistently detect parasitaemia, with positive detection ranging from 4/13 to 12/16 of sampled time points per animal.

Diminazene aceturate was administered on day 28 in all four cattle. In total 24 h after treatment, the BCT and the DNA PCR tests yielded negative results. The 7SL sRNA signal remained detectable until 48 h after treatment in three cattle and until 72 h in one, after which it was negative.

Following *T. congolense* IL3000 infection, the 7SL sRNA RT-qPCR detected infection earlier than BCT, and on the same day or in one calf a day earlier than the TCS PCR. The 7SL sRNA signal remained present throughout the infection but was no longer detectable from 48 to 72 h after treatment, making the RT-qPCR based method the most sensitive out of the three methods.

### Signal Decay

The 7SL sRNA detection data suggests that it is linked to active infection, because the signal decays post-treatment. The rate of this decay is important to understand, as it provides information on how well 7SL sRNA can directly link to active infection (potentially important in, for example, assessing clinical efficacy of drugs or vaccines), and also informs on how a positive signal can be interpreted in an animal. In order to study the dynamics of the 7SL sRNA signal decay after treatment, Holstein-Friesian calves were experimentally infected with *T. congolense* and *T. vivax* (*n* = 15 per species) and treated at the peak of infection (determined as a parasitaemia score ≧ 4; equivalent to 1 × 10^4^–5 × 10^5^ parasites/mL) with an experimental trypanocidal drug. Calves infected with each of the trypanosome species were distributed in three groups (*n* = 5 per group) depending on the drug dosage they received (0.5× , 1× , and 2× the minimal effective treatment dose for the drug used), and the infection levels monitored 24-, 48-, 72- and 96-h post-treatment. Parasite presence was detected by the BCT in all experimentally infected calves, confirming that infection had been successfully established.

### 7SL sRNA Signal Decay in *T. congolense* Infections

**Figure 3:**
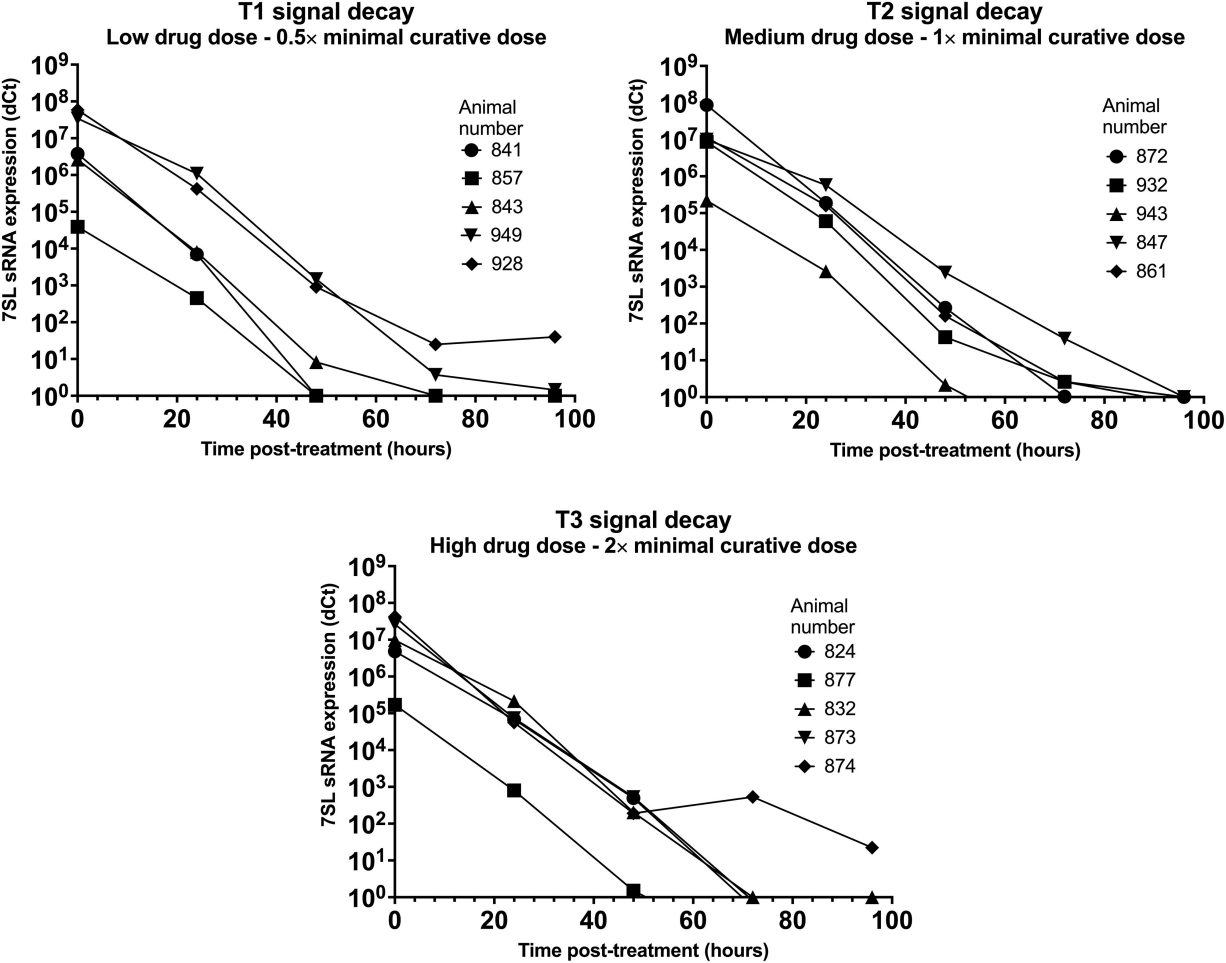
Decay of 7SL sRNA signal in *T. congolense* infected cattle after trypanocide treatment. Cattle (Holstein-Friesian, *n* = 5 per treatment group) were infected with *T. congolense* Kont2/133 (day 0) at peak parasitaemia (1 × 10^4^-5 × 10^5^ trypanosomes/ml), cattle were treated with a low (equivalent to 0.5 × minimal effective dose), medium (1×) or high dose (2×) of an experimental trypanocide. Relative expression of 7SL sRNA by RT-qPCR is shown with a black line and was calculated by normalizing to an uninfected control animal.

Parasite presence in cattle from group T1 (low drug dose - 0.5× minimal curative dose; note, the dose received is the same for the following *T. vivax* infections) was detected using BCT at day 7 post-infection, while the 7SL sRNA signal was first detected at day 3 in two animals, and at day 7 in the remaining three (data prior to drug treatment not presented). Trypanocidal treatment was administered on day 11 post-infection, and 24 h post-treatment parasite presence in blood could not be detected by the BCT. The 7SL sRNA signal decreased 24 hours post-treatment and disappeared from 48 to 96 h after treatment (Figure 3), except in Animal 928, in which the 7SL sRNA signal was 1,477,780-fold lower but had not disappeared by 96 h post-treatment, indicating that the small RNA was still present.

Among cattle in group T2 (medium drug dose – 1× minimal curative dose), parasite presence was first detected using BCT 7 days after infection. Earlier detection was achieved using the 7SL sRNA RT-qPCR which was positive 3 days after infection in four of the five cattle. Trypanocidal treatment was administered on day 8 or 11 after infection, and 24 h post-treatment parasite presence in blood could not be detected by the BCT. The 7SL sRNA signal was reduced at 24 h post-treatment and completely disappeared by 48 to 96 h after treatment in all animals (Figure 3).

In group T3 (high drug dose – 2× minimal curative dose), parasite presence was first detected using BCT 7 days after infection while the 7SL sRNA signal was first detected 3 days after infection. Trypanocidal treatment was administered on day 8 or 11 after infection. From 24 h post-treatment, parasite presence in blood was not detected using BCT. The 7SL sRNA signal was lower 24 h post-treatment and completely disappeared from 48 to 96 h after treatment (Figure 3). In animal 874, the 7SL sRNA signal had been reduced by 1,896,621-fold but had not completely disappeared by 96 h post-treatment, indicating that the small RNA was still present.

The 7SL sRNA signal was reduced after treatment in all three groups, suggesting that 7SL sRNA presence in circulation is linked to active infection. No apparent differences in signal decay were observed between the three treatment groups. However, two calves from T1 (857 and 949) later relapsed after treatment, with blood parasitaemia detected by BCT on day 25 post-infection. No indication of this relapse was given by the 7SL sRNA signal, but the last sample that was available to test by 7SL sRNA was 10 days before parasite presence was detected. Signal decay time varied between 48 to 96 h in all three groups, with the exception of calves 928 (T1) and 874 (T3) for which the 7SL sRNA signal remained present within the tested time frame. This increased decay time may be due to animal pharmacokinetic differences or potentially a more drug-refractory subpopulation of parasites being present in the animals at time of treatment [for example, parasites potentially occupying drug-inaccessible tissue niches (41, 42)].

### 7SL sRNA Signal Decay in *T. vivax* Infections

**Figure 4:**
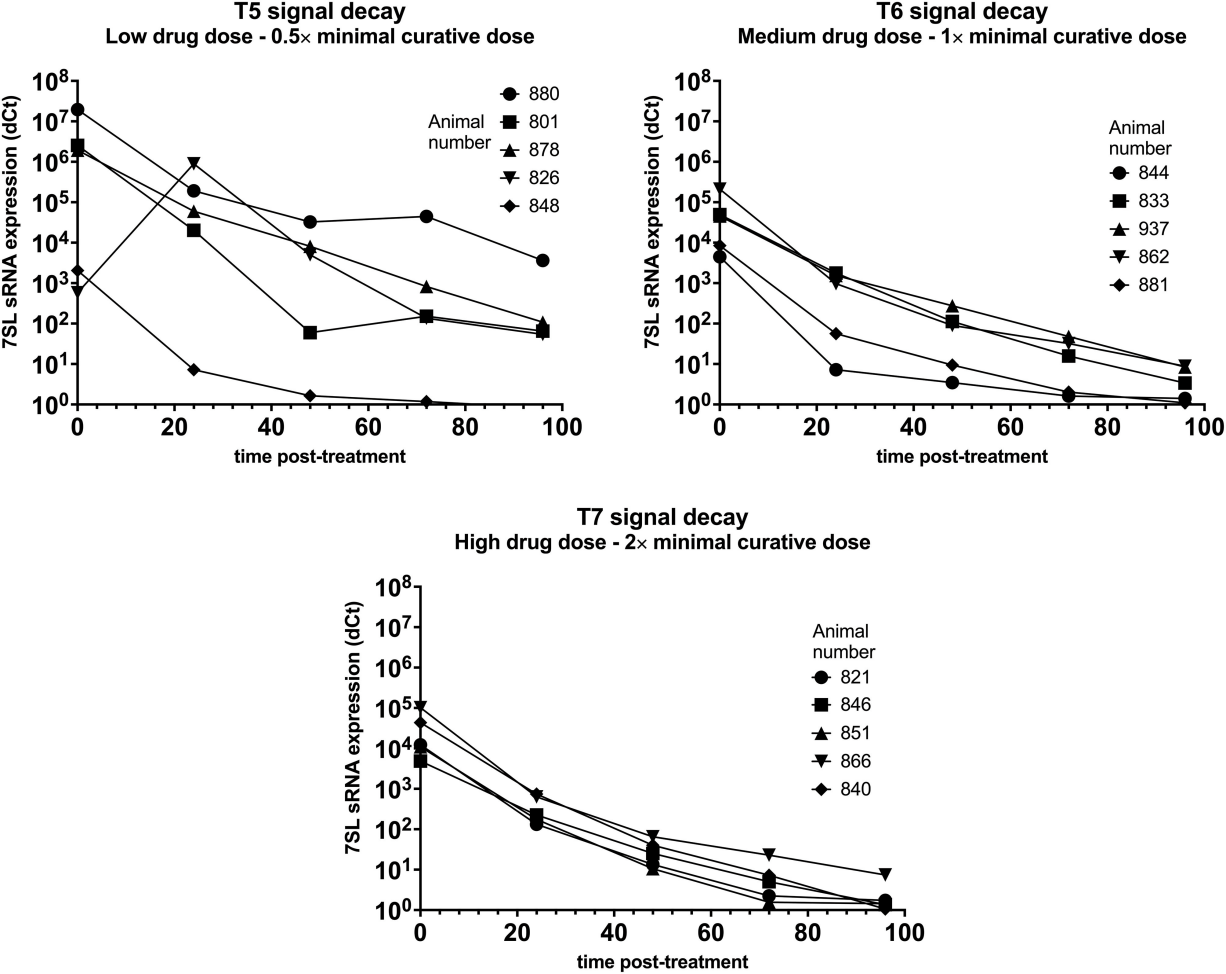
Decay of 7SL sRNA signal in *T. vivax* infected cattle after trypanocide treatment. Cattle (Holstein-Friesian, *n* = 5 per treatment group) were infected with *T. vivax* STIB 719 (day 0) at peak parasitaemia (1 × 10^4^-5 × 10^5^ trypanosomes/ml), cattle were treated with a low (equivalent to 0.5 × minimal effective dose), medium (1×) or high dose (2×) of an experimental trypanocide. Relative expression of 7SL sRNA by RT-qPCR is shown with a black line and was calculated by normalizing to an uninfected control.

Parasite presence in cattle from group T5 (low drug dose – 0.5× minimal curative dose) was detected using BCT at day 6 post-infection, while the 7SL sRNA signal was detected from day 3 post-infection. Trypanocidal treatment was administered on day 7 post-infection, and 24 h post-treatment parasite presence in blood could not be detected by the BCT. The 7SL sRNA signal decreased 24 h post-treatment and was not detected 96 h post-treatment in one calf. In the remaining four calves, the 7SL sRNA signal was still present but was reduced by an average of 14,812-fold 96 h after treatment (Figure 4).

Among cattle in group T6 (medium drug dose – 1× minimal curative dose), parasite presence was first detected using BCT 6 or 7 days after infection. Earlier detection was achieved using the 7SL sRNA RT-qPCR which was positive 3 days after infection in 4 of the 5 cattle. Trypanocidal treatment was administered on day 7 or 8 after infection. At 24 h post-treatment parasite presence in blood could not be detected by the BCT. The 7SL sRNA signal was reduced at 24 h post-treatment and completely disappeared by 96 h after treatment in two calves. In the remaining three calves the 7SL sRNA signal was detectable but had been reduced by 14,630-fold 96 h post-treatment (Figure 4).

In group T7 (high drug dose – 2× minimal curative dose), parasite presence was first detected using BCT 7 days after infection while the 7SL sRNA signal was first detected from 3 or 6 days after infection. Trypanocidal treatment was administered on day 7 after infection. At 24 h post-treatment, parasite presence in blood was not detected using BCT. The 7SL sRNA signal was lower 24 h post-treatment and completely disappeared at 72 to 96 h after treatment (Figure 4). In calf 866, the 7SL sRNA signal was reduced 13,777-fold 96 h post-treatment but remained detectable.

7SL sRNA signal was reduced after treatment in all three groups, suggesting that 7SL sRNA presence in circulation is linked to active infection. However, for these *T. vivax* infected animals, there were clear differences in decay dynamics between the three groups, suggesting a correlation between a higher drug dose and a faster signal decay – this is likely due to the low dose of drugs not effectively clearing all parasites by 96 h and the high dose clearing parasites quicker than the medium dose – however, it is worth noting that no relapses were observed in *T. vivax* infected animals after the 96 h post-treatment period.

### Cross-Reaction With *T. theileri* and Conservation of 7SL SRNA Sequence Within Species

**Figure 5:**
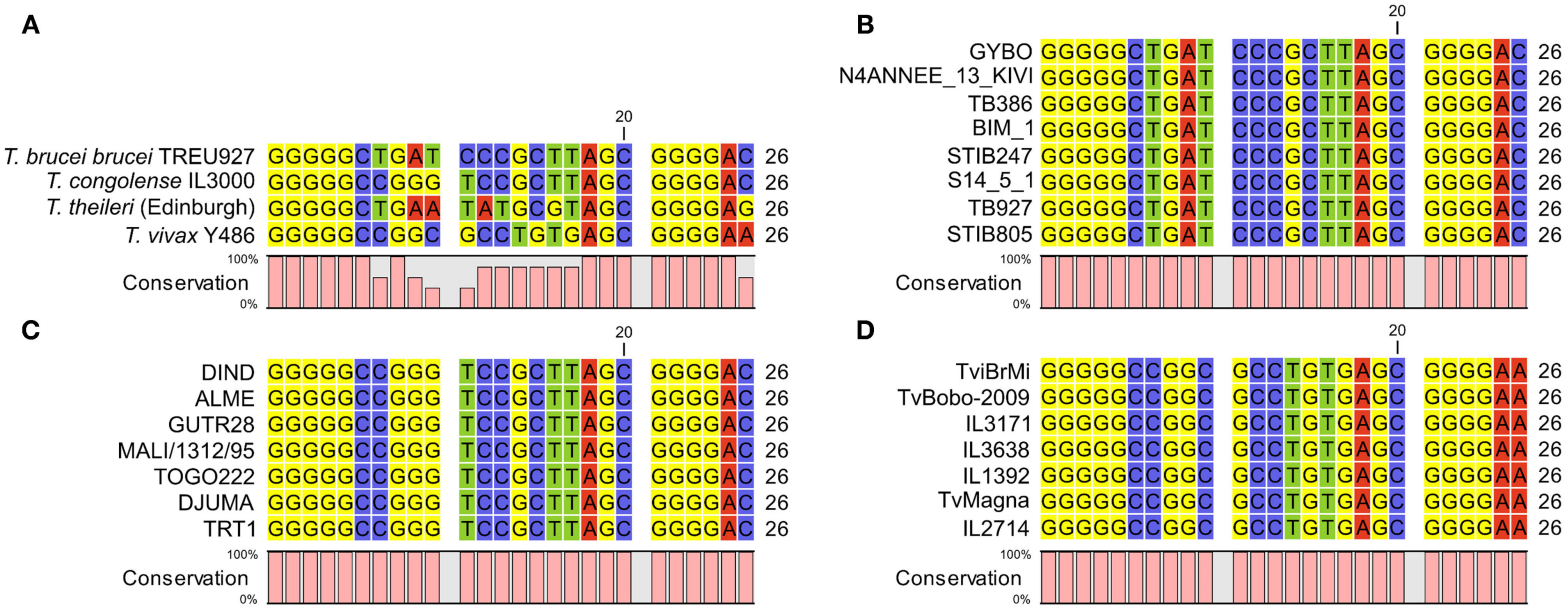
**(A)** alignment of 7SL sRNA sequences from African trypanosomes and the commensal *Trypanosoma theileri*. Consistent with the African trypanosomes previously analyzed, the *T. theileri* 7SL sRNA sequence exhibits highly conserved flanking regions with a variable internal sequence, which importantly does not match that of the other trypanosome species. **(B)** 7SL sRNA sequences alignment in representative *T. brucei* subspecies isolates (from a total of 88 analyzed). Genome data were obtained from a previous study (34) (ENA accession: PRJEB2486) or from TriTrypDB. Strain information: GYBO: *T. b. rhodesiense*, isolated in 1984 in Tanzania; N4ANNEE_13_KIVI: *T. b. gambiense* group I, 2002, Guinea; TB386: *T. b. gambiense* group II, 1978, Côte d'Ivoire; BIM_1: *T. b. gambiense* group I, 1975, Cameroon; STIB247: *T. b. brucei*, 1971, Tanzania; S14_5_1: *T. b. gambiense* group I, 2002, Côte d'Ivoire; TB927: *T. b. brucei*, 1969 Kenya; STIB805: *T. b. evansi*, 1985, China. **(C)** 7SL sRNA sequence alignments in *T. congolense* isolates. Data from 55 genomes were obtained from a previous study (35) (ENA accession: PRJEB15251), and a representative subset is shown. Strain information (Savannah subtype unless stated): DIND: Forest subtype, 1986, Burkina Faso; ALME: 2005, Cameroon; GUTR28: 1975, The Gambia; MALI/1312/95: 1995, Mali; TOGO222: 2014, Togo; DJUMA: 1988, Congo; TRT1: 1996, Zambia. **(D)** 7SL sRNA sequence alignments in *T. vivax* isolates. Data from 27 genomes were obtained from a previous study (36) (ENA accession number: PRJNA486085) and a representative subset is shown. Strain information: TviBrMi: 1999, Brazil; TvBobo-2009: 2009, Burkina Faso; IL3171: year unknown, The Gambia; IL3638: 1990, Côte d'Ivoire; IL1392: 1981, Nigeria; TvMagna: 2011, Togo; IL2714: 1969, Uganda.

Analysis of the *T. theileri* genome (21) indicates that there is an ortholog of the 7SL sRNA locus (although it is currently unannotated as such). Although it contains sufficient sequence differences to suggest that the assays for *T. brucei, T. congolense and T. vivax* would be unlikely to cross-react (Figure 5A), it is still important to formally show this given the broad distribution of *T. theileri* and frequency of co-infections. To test for the possibility of cross-reactions between the assays designed for detection of *T. brucei, T. congolense* and *T. vivax* with *T. theileri*, samples from experimental infections were analyzed. Holstein-Friesian calves (*n* = 8) were infected with 1 × 10^7^ *T. theileri* parasites (Edinburgh strain) and infections followed for 8 weeks, and successful establishment of the infection confirmed by nested PCR targeting the *T. theileri* β-α tubulin intergenic sequence or the SSU rRNA gene. Samples remained PCR positive 3- and 8-weeks post-infection. Uninfected sera samples as well as samples from the peak of parasitaemia (3 weeks post-infection) and cryptic infection (8 weeks post-infection) were tested by RT-qPCR using the *T. brucei, T. congolense* and *T.vivax* 7SL sRNA assays. The 7SL sRNA (*T. brucei, T. congolense and T. vivax* specific assays) was undetectable pre-infection as well as 3- and 8-weeks post-infection in all calves (Ct = 40 in all samples). These results indicate that the 7SL sRNA assays designed to detect presence of *T. brucei, T. congolense* or *T. vivax* do not cross-react with *T. theileri*.

A further remaining question was how conserved the 7SL sRNA sequence is within each species, which is important for assessing confidence in how well the 7SL sRNA assay will perform on samples from different geographical areas with genotypically different circulating isolates; this is particularly of interest for *T. congolense*, where it is well established that there are three genetically distinct subtypes, Savannah, Forest and Kilifi (1, 43). We compared the 7SL sRNA sequences in multiple diverse strains extracted from data generated in previous population genomic studies of *T. brucei* (34) (*n* = 85), *T. congolense* (35) (*n* = 51) and *T. vivax* (36) (*n* = 27). Within *T. brucei*, this diversity spanned isolates representing *T. b. brucei, T. b. rhodesiense, T. b. gambiense* and *T. b. evansi*, for *T. congolense* this included multiple geographically diverse isolates of *T. congolense* Savannah and the currently available *T. congolense* Forest genomes, and for *T. vivax* this included multiple geographically diverse isolates (including a South American isolate). Therefore, this dataset is as representative as is currently possible of the diversity present within each species. The 7SL sRNA sequence was 100% conserved across all isolates in all three species (Figures 5B–D). This indicates that, as far as current knowledge allows us to assess, the assay should be equally effective in detecting 7SL sRNA for all strains within *T. brucei, T. congolense* and *T. vivax*.

### Sensitivity and Specificity

Determining the sensitivity and specificity of a diagnostic test is essential to evaluate the accuracy of a test and enable comparison with other existing methods of diagnosis. Although the 7SL sRNA diagnostic is based on detecting the presence or absence of the molecule, the RT-qPCR technique may display low levels of background signal when negative samples are tested. Considering this, it is especially important for application of the test to field samples to establish a cut-off point that would rule that a test is positive (infected) or negative (uninfected).

Receiver Operating Characteristic (ROC) analysis is a graphical representation for the ability of a test to distinguish between infected and uninfected patients. The ROC analysis calculates the sensitivity and specificity of the diagnostic test for different cut-off values, aiding in deciding the cut-off that maximizes sensitivity and specificity. To determine the 7SL sRNA RT-qPCR sensitivity and specificity for each of the AT-causing trypanosome species, test results from all the available experimental samples (Roslin and Clinvet samples in this study, and those previously published by Chiweshe et al. in 2019 (29); *n* = 211 for *T. brucei, n* = 276 for *T. congolense*, and *n* = 150 for *T. vivax*) were classified as uninfected and infected samples and ROC analysis performed. Samples were classified as definitively uninfected if they were taken before parasite inoculation, samples were considered infected from the inoculation time-point up until treatment.

The cut-off point decision was based on optimisation of sensitivity and specificity of the test. However, this cut-off can be lowered if high sensitivity is desired (at a cost of lower specificity) or raised if high specificity is needed (at a cost of lower sensitivity). Besides the calculation of sensitivity and specificity, the ROC analysis allows for the calculation of the area under the curve (AUC). The AUC reflects how good the test is at distinguishing between presence and absence of disease. This measure is independent of prevalence and summarizes the discriminative ability of the test across the full range of cut-offs. Generally, it is accepted that a perfect test would have an AUC of 1, whereas an ineffective test would have an AUC of 0.5. The closer to 1 the AUC is, the better the test: AUC ≥ 0.9 high accuracy, AUC ≥ 0.7 moderate accuracy, AUC ≥ 0.5 low accuracy.

### *T. brucei* ROC

**Figure 6:**
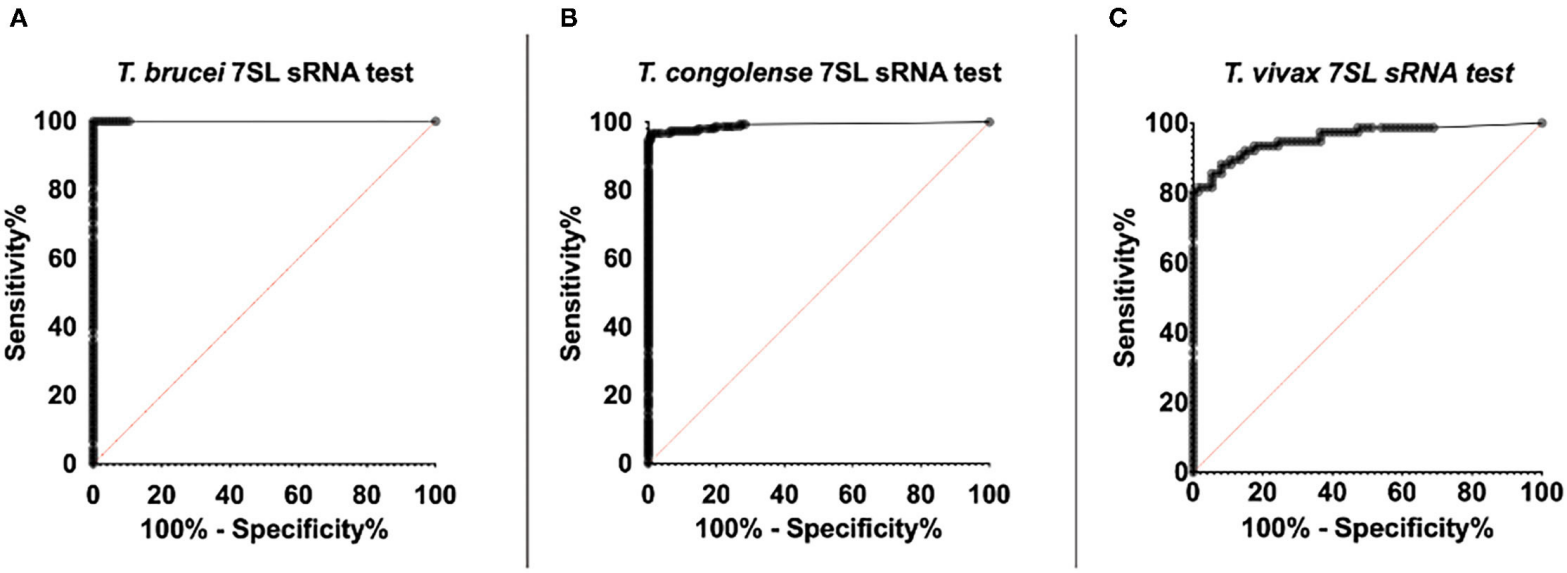
ROC curve of RT-qPCR detection of trypanosome specific 7SL sRNA. ROC analysis was performed using the Wilson/Brown method. The ROC curve of the 7SL sRNA test is represented by the series of black dots. The red diagonal line works as a reference and represents random chance, the characteristics of a test that is not useful for in detecting infection (AUC = 0.5). **(A)** 107 *T. brucei* Antat 1.1 infected samples and 104 uninfected samples were included in the analysis (AUC=1). **(B)** 153 *T. congolense* (IL3000 or Kont2/133) infected samples and 123 uninfected samples were included in the analysis (AUC = 0.99). **(C)** 76 *T. vivax* STIB 719 infected samples and 74 uninfected samples were included in the analysis (AUC = 0.96).

For the 7SL sRNA *T. brucei* diagnostic test, 107 infected samples and 104 uninfected samples were included in the ROC analysis (Figure 6A). The Ct determined to maximize sensitivity and specificity was found to be 35.71 by both the (0, 1) method and Youden's index. A cut-off of 35.71 results in a test with 100% sensitivity (95% CI, 96.44–100%) and 100% specificity (95% CI, 96.53–100%). The calculated AUC for the analysis was 1 (95% CI, 1–1) indicating the high accuracy of this diagnostic method.

### *T. congolense* ROC

For the 7SL sRNA *T. congolense* test, 153 infected samples and 123 uninfected samples were included in the ROC analysis (Figure 6B). The Ct determined to optimize sensitivity and specificity was 35.28 by both the (0, 1) method and Youden's index. A cut-off of 35.28 has an associated sensitivity of 96.73% (95% CI, 95.54–99.96%) and 99.19% specificity (95% CI, 92.58–99.60%). The AUC value of this diagnostic method was calculated to be 0.99 (95% CI, 0.98–1), indicating the high ability of the test to correctly identify presence and absence of disease.

### *T. vivax* ROC

For the 7SL sRNA *T. vivax* test, 76 infected samples and 74 uninfected samples were included in the ROC analysis (Figure 6C). The cut-off (Ct) determined to optimize sensitivity and specificity was 31.38 by both the (0, 1) method and Youden's index. A cut-off of 31.38 has an associated sensitivity of 93.42% (95% CI, 85.51–97.16% %) and 82.43% specificity (95% CI, 72.23–89.44% %). The AUC value of this diagnostic method was calculated to be 0.96 (95% CI, 0.93 to 0.99), indicating the high ability of the test to correctly identify presence and absence of disease.

## Discussion

Here, we have demonstrated the comparative accuracy of 7SL sRNA as a diagnostic marker for AT infection in cattle compared with other available diagnostic tests. Detection of 7SL sRNA by RT-qPCR allowed earlier diagnosis than both the BCT microscopy techniques and the highly sensitive DNA PCR targeting multi-copy satellite repeats. The 7SL sRNA signal could be detected throughout the duration of the infection, even in periods when parasitaemia was sub-patent. These results suggest that 7SL sRNA is a sensitive marker of early infection compared with the current gold-standard tests; microscopy and DNA PCR. After treatment with the trypanocide diminazene aceturate, the 7SL sRNA signal, in most cases, was not detectable 48-96 h after treatment. For future application of the 7SL sRNA diagnostic test to field samples, a test cut-off was determined performing a ROC analysis to maximize specificity and sensitivity. The ROC analysis demonstrated the high accuracy of the three species-specific trypanosome tests and determined the sensitivity of the three tests to be above 93% and specificity of the three tests to be above 82%.

Following infection with *T. brucei* AnTat 1.1 and then treatment with diminazene aceturate, parasites were not detected in blood using BCT, and TBR-PCR results were negative 24 h after treatment. The 7SL sRNA signal showed substantially decreased expression after treatment compared to pre-treatment values, but while decreasing in all animals the signal did remain detectable at the end of the time frame analyzed (96 h post-treatment). We considered various hypotheses to explain this result. First, we reasoned that the 7SL sRNA might have a long life in biofluids, which would be consistent with the high stability reported by Verney 2020. Nevertheless, in cattle infected with *T. congolense* IL3000 and treated with the same drug, the 7SL sRNA signal was undetectable 48–72 h after treatment. Given the high sensitivity of the test and the fact that the 7SL sRNA is secreted/excreted at high amounts in blood, we hypothesized that the result observed could indicate that diminazene aceturate at 7 mg/kg cleared *T. brucei* AnTat 1.1 less rapidly at the dosage applied than it does *T. congolense* IL3000. There are currently no reports of *T. brucei* AnTat 1.1 being less sensitive to diminazene aceturate than other *T. brucei* strains, and little is known about the drug sensitivity profile of this isolate *in vivo*. However, it has been shown that *T. brucei* AnTat 1.1. has a substantially lower sensitivity to isometamidium chloride than *T. congolense* IL3000 during *in vitro* assays (IC_50_ values of 9.24 and 0.56, respectively) (44). Given that diminazene aceturate and isometamidium chloride are closely related, and that cross-resistance between these compounds is known to occur in both field and experimental conditions (3, 45–47), the relative susceptibility of *T. brucei* AnTat 1.1 is worthy of further investigation. It is also possible that in *T. brucei*, extravascular subpopulations [e.g. as have been shown in mice and humans to occur in the adipose and skin (41, 42, 48)] are relatively protected from circulating drugs, and either take longer to cure as a consequence, or these subpopulations potentially serve as reservoirs for relapse of infection. Note that *T. congolense* is considered an intravascular parasite (49, 50). Ideally, tissue samples would have been taken post-mortem to assess if 7SL sRNA signal remained that would indicate either extravascular foci or vascular adherent cells, or the cattle would have been monitored for longer periods after treatment to assess possible relapse, which would have enabled assessment of whether this isolate is less sensitive to diminazene aceturate, or if diminazene aceturate takes longer to clear *T. brucei* parasites from cattle than *T. congolense*- however, neither of these options were possible within the scope of this study.

In cattle infected with *T. congolense* IL3000, treatment with diminazene aceturate prompted a rapid decay of the 7SL sRNA signal which was not detectable 48–72 h post-treatment, suggesting that the 7SL sRNA is an accurate marker of active infection. Similarly, in cattle infected with *T. congolense* Kont2/133 and then treated with an experimental trypanocidal compound, 7SL sRNA was undetectable 48–96 h after treatment. No apparent differences were observed between the signal decay dynamics of the three treatment groups with differing doses of drug. However, two cattle from the low drug dose group (T1; 0.5× minimal curative dose) relapsed and presented parasitaemia in blood 10 days after the last 7SL sRNA tested time point. These results suggest that 7SL sRNA is an accurate marker of active infection, but extended monitoring (beyond 96 h) may be required to detect potential treatment failures. In our previous publication (29) we showed how 7SL sRNA can be more effective than microscopy for early detection of relapses - however, samples were not available in the current study to further examine this property.

Cattle infected with *T. vivax* STIB719 and treated with an experimental trypanocide drug presented different 7SL sRNA signal decay dynamics depending on the dose administered. In cattle treated with the low drug dose (T5; 0.5× minimal curative dose), 7SL sRNA signal declined but remained detectable 96 h after treatment in 4 of the 5 animals. In groups T6 and T7, medium and high drug dose, respectively, 7SL sRNA signal decay time appeared shorter, although the signal decay was the most rapid in the high drug dose group, suggesting a correlation between drug dose administered and signal decay time. None of the cattle relapsed during follow up after the 96-h post-treatment period, suggesting that the drug used may clear *T. vivax* more slowly than it does *T. congolense*.

These data illustrate the potential utility of the 7SL RNA assay in terms of informing on rapidity of parasite killing and clearance from circulation. In combination with the data presented in Chiweshe et al. (29), where it was demonstrated that the 7SL RNA assay was more effective at detecting post-treatment relapses than microscopy, this indicates that the 7SL RNA assay can be a powerful tool for detecting active infection, and may be useful in particular for assessing efficacy of drug or vaccine candidates, where with current methods long term follow-up is necessary to ensure parasite clearance has been achieved.

In the development of new diagnostics, studying the accuracy of the test is essential to evaluate its performance and compare it with existing diagnostic tests. The 7SL sRNA RT-qPCR for *T. brucei, T. congolense* and *T. vivax* exhibited sensitivity and specificity above 93 and 82% in the ROC analyses, respectively, and an AUC value above 0.96. These results suggest the test is highly accurate. The sensitivity and specificity of tests for AT diagnosis is under-reported, probably due to high variation in their performance between acute infection and chronic disease. Instead, most diagnostic studies report a threshold of parasite concentration in blood that the particular method is able to detect. The BCT has a threshold of detection estimated to be 250 trypanosomes/mL and, thus, can be highly sensitive for acute infections (sensitivity ~80%) but performs poorly in chronic infections (sensitivity <10%) (51). Specificity of the BCT is 100% for detection of *Trypanosoma* but requires an expertly trained eye to distinguish between trypanosome species (14, 51). There are relatively few reports (16, 52, 53) on the sensitivity and specificity of the TCS and TBR PCRs for diagnosis of *T. brucei* and *T. congolense* infections in cattle. In these publications, the sensitivity is reported to be between 0.1–10 trypanosomes/mL; the exact sensitivity depends on whether concentration of the trypanosome fraction has been undertaken before PCR amplification and the method used for sampling and DNA extraction. The OIE warns about the high false negative rate of PCR when parasitaemia is <1 trypanosome/mL, which is frequent in chronic infections (14). However, it should be noted that this detection threshold is not due to a lack of PCR sensitivity, but is due to absence of parasites and therefore parasite DNA in samples when parasitaemia levels and sampling volumes are very low – in contrast, a target such as the 7SL sRNA is excreted/secreted in large numbers by each cell, meaning that an assay targeting 7SL sRNA is not reliant upon the test sample containing a parasite.

We have also demonstrated that the 7SL sRNA RT-qPCR assays for *T. brucei, T. congolense* and *T. vivax* do not cross-react with the commensal *T. theileri*, which is ubiquitous in cattle worldwide. Additionally, we undertook a comprehensive analysis of 7SL sRNA sequences across multiple genomes of *T. brucei, T. congolense* and *T. vivax*, incorporating as much as is currently possible the diversity within these species. These analyses demonstrated that the 7SL sRNA sequence was 100% conserved within species, indicating that the 7SL sRNA assays should be able to equally accurately detect signal from any strain within the species – this was important to assess given the known genetic diversity within species (1, 34–36, 43, 54). While we could not assess conservation across the full spectrum of trypanosome diversity due to lack of genome availability (e.g. *T. vivax*-like strains (55, 56) – currently not known to cause disease in livestock), for the species (and strains/subspecies/subtypes) that are known to cause the majority of disease in livestock (i.e. *T. brucei, T. congolense* and *T. vivax*), we would therefore be confident that the 7SL sRNA sequence is conserved and the assay would be applicable.

Although we have shown that the 7SL sRNA RT-qPCR is able to detect infection in periods of sub-patent parasitaemia, it would be of particular interest to test the accuracy of the diagnostic in chronic cases of AT. Ideally, this would be from long-term longitudinal studies under controlled experimental conditions that would minimize confounding effects of co-infections, treatments and other factors such as nutrition, as well as from well characterized field samples from trypanosome endemic areas, such that the performance of the assay can be assessed across differing infection dynamics and clinical settings, and its future utility defined.

## Conclusions and Future Directions

The characteristics described in this article make the 7SL sRNA RT-qPCR an ideal diagnostic tool for assessing active infection, with particularly obvious utility in drug or vaccine clinical trials - the high sensitivity and accuracy of the test enables early and accurate detection of treatment failure, a feature that is especially desirable given the costs involved in the clinical assessment of new treatments in cattle, particularly in LMIC settings. Additionally, the assay would potentially add value to epidemiological studies, for which one of the most widely used tests (ELISA) does not discriminate between active infection and exposure (this particularly relevant to hyperendemic areas with frequent treatment and/or reinfection), and microscopy techniques have well established lower sensitivity. Although some estimates are available for the extent of AT disease across sub-Saharan Africa, as well as some regions in South America that are experiencing an increase in cases, accurate diagnostics would represent the first step toward verifying the true magnitude of the issue and aid in establishing better control and elimination measures. Indeed, assessing the current scale of trypanosomiasis within any country is the first step of the five step Progressive Control Pathway for AT proposed by the FAO (18), and the 7SL sRNA based test could play a valuable role in generating accurate relevant information.

Currently diagnosis of AT requires relatively costly equipment and requires a degree of expert interpretation. Veterinary services are expensive and often not accessible to or are not accessed by many livestock owners in rural or remote regions of sub-Saharan Africa. This means that diagnosis is often subjective and based on clinical signs that are not pathognomonic, resulting in farmers often misdiagnosing AT and/or administering unnecessary drug doses for treatment (19). It would be extremely desirable to develop a rapid and cost-effective diagnostic test that can be used in a field setting before deciding on a trypanocidal treatment. A rapid diagnostic test has been developed (VeryDIAG) adapting an indirect ELISA to a lateral flow test strip. VeryDIAG can diagnose and discriminate between *T. congolense* and *T. vivax* infections (17). However, as it is antibody based, this test is not able to distinguish between active and past infections, limiting its use in areas with high prevalence of the disease, in which farmers routinely administer trypanocidal treatments and where there are often very frequent reinfections. Given the few drugs available for AT, and with only one prospective new drug on the horizon (57), diagnosis of active infection could be an essential future asset in terms of maximizing the usable lifespan of these compounds – targeting accurate treatment to those animals that need it is likely to be essential in minimizing the emergence and impact of drug resistance (3, 18, 19).

The 7SL sRNA has proved to be a powerful potential diagnostic marker of AT with many desirable attributes: early infection detection, continued signal even in the absence of detectable parasitaemia in blood, discrimination between infected and treated animals, a high copy number of the target molecule in biofluids, long-term stability at room temperature, and the ability to distinguish between *T. brucei, T. congolense* and *T. vivax*—the three trypanosome species that cause AT in cattle. These characteristics make 7SL sRNA an ideal candidate on which to base a rapid diagnostic test that could be used in a field setting. Indeed, other small RNAs are being developed into rapid diagnostic markers using novel assays and platforms [reviewed in (58, 59)]. For instance, Tran and Phung developed a LAMP assay able to detect *Fasciola hepatica* derived microRNAs in bovine serum samples (60). This assay has the advantage of not requiring an initial heating step and providing a SyBr green based visual readout, making it potentially easier to perform in a field setting. Although the number of samples the study evaluated was low, there are indications of the potential high sensitivity of this method, reported by the authors to be able to detect 1 zmol of the target miRNA (60). The use of Cas13 in the detection of small RNAs has also been indicated to achieve rapid and accurate diagnosis (59). Cas13 based detection was initially designed to diagnose RNA virus infections. Cas13a is guided by the crRNA to its ssRNA target, when Cas13a binds its target it cleaves it, but also cleaves other surrounding ssRNAs indiscriminately. The addition of a cleavage activated reporter in the reaction mix provides a simple readout of target detection (61). This method of RNA detection has been adapted to detect tumor microRNAs by using a microfluidic electrochemical biosensor platform. This platform has the advantage of being point-of-care and having a processing time of less than 4 h, and a limit of detection of 10 pM (62).

The challenge for the application of small RNA detection protocols for AT will be to reduce reagent costs to the level where diagnosing infection becomes financially feasible for end users (as a comparison, treatment with diminazene aceturate costs farmers approximately $1), as well as meeting requirements for a rapid, simple, easy to perform and interpret test, while maintaining the sensitivity and specificity observed under experimental conditions. While this clearly presents a challenge, the 7SL sRNA has many of the requisite properties for sensitively and specifically detecting active infection, and this, combined with technological advances in diagnostic platforms for small RNAs, provides cause for optimism that the 7SL sRNA assay may in the future provide a long-needed diagnostic tool for animal trypanosomiasis.

## Data Availability Statement

The original contributions presented in the study are included in the article/supplementary material, further inquiries can be directed to the corresponding author/s.

## Ethics Statement

The animal studies were reviewed and approved by Roslin Institute Animal Welfare and Ethical Review Board and Clinvet Institutional Animal Care and Use Committee.

## Author Contributions

MC, KF-C, JM, FG, and LM: conception or design of the study. MC, EW, EP, PS, JL-V, MP, KM, FE-A, AE, KF-C, JM, FG, and LM: acquisition, analysis or interpretation of the data. MC, EW, EP, PS, JL-V, MP, KM, FE-A, AE, KF-C, JM, FG, and LM: writing of the manuscript. All authors contributed to the article and approved the submitted version.

## Funding

MC, KF-C, JM, FG, and LM were supported by funding from Roslin Technologies Limited. KM received support from the UK Biotechnology and Biological Sciences Research Council (BBSRC; grant number BB/L02442X/1) and the Wellcome Trust (103740/Z/14/Z). The Roslin Institute and MC, EP, PS, FG, and LM were funded by the BBSRC (BS/E/D/20002173). Serum samples from Site 2 used in this study derived from a study commissioned by Global Alliance for Livestock and Veterinary Medicine (GALVmed) with funding from Bill & Melinda Gates Foundation grant OPP1200611 and UK Aid grant 300504.

## Conflict of Interest

EW, KF-C, and JM were employed by the company Roslin Technologies Limited. AE and FE-A were employed by the company Clinvet Morocco. The remaining authors declare that the research was conducted in the absence of any commercial or financial relationships that could be construed as a potential conflict of interest.

## Publisher's Note

All claims expressed in this article are solely those of the authors and do not necessarily represent those of their affiliated organizations, or those of the publisher, the editors and the reviewers. Any product that may be evaluated in this article, or claim that may be made by its manufacturer, is not guaranteed or endorsed by the publisher.
